# Situation analysis of urogenital bilharzia in West Africa (2010-2021) and control strategies and prospects: systematic review and meta-analysis

**DOI:** 10.11604/pamj.2023.44.35.33766

**Published:** 2023-01-18

**Authors:** Josias Olutobi Ahamide, Charles Sossa, Yolande Sissinto, Virginie Mongbo, Victorien Dougnon, Boris Legba, Edgard-Marius Ouendo

**Affiliations:** 1 Institut Régional de Santé Publique, Université d'Abomey-Calavi, Abomey-Calavi, Bénin,; 2 Laboratoire de Parasitologie, Faculté des Sciences de la Santé, Université d'Abomey-Calavi, Abomey-Calavi, Bénin,; 3 Laboratory of Biology and Molecular Typing in Microbiology, Research Unit of Applied Microbiology and Pharmacology of Naturals Substances, University of Abomey-Calavi, Abomey-Calavi, Bénin

**Keywords:** Bilharzia, strategies, prevalence, systematic review, meta-analysis, West Africa

## Abstract

Schistosomes are parasitic diseases caused by flatworms (schistosomes or bilharzia), transmitted in the urine or in the faeces, and involving intermediate hosts (freshwater molluscs). Their recrudescence in endemic areas is no longer in question and remains a crucial public health problem in the world in general and in West Africa in particular. In order to eradicate bilharzia, many control strategies and policies have been implemented on both sides. The objective of this systematic literature review is to synthesize the existing evidence on control strategies implemented by West African countries. To achieve this, data were collected from PubMed, Direct Science, Web of Sciences, Google Scholar, PloS and Banque de Données de Santé Publique (BDSP), using appropriate keywords. Academic articles and theses written in French or English that evaluated the analysis of a bilharzia situation in West Africa were selected. Sixteen scientific papers were selected for the study, ten of which were used for a meta-analysis. The systematic review revealed that bilharzia is still an endemic disease in West Africa. Clearly, it continues to wreak havoc on the population, especially among school children. Rural areas are the most affected by the disease. Strategies to control bilharzia are based on preventive and curative treatment of the infection with chemotherapy and vector control of soil molluscs (host and vector of bilharzia eggs). Praziquantel is the main known antibilharzian. Also, the species most frequently found in analyses are S. haematobiumand S. mansonii. This review has allowed to evaluate the control strategies carried out and to deduce the strengths and weaknesses, in order to define the perspectives for the efficiency of the anti-bilharzia control for the eradication of bilharzia in the endemic zones of West Africa.

## Introduction

In sub-Saharan Africa, bilharzia or schistosomiasis is the second most prevalent and important neglected tropical disease in terms of public health, after hookworm [1-3]. The number of people exposed worldwide is estimated at 600 million, with more than 200 million people infected and nearly 280,000 deaths each year, 97% of which occur in Africa, south of the Sahara [2,3]. Schistosomiasis accounts for 1.9 million disability-adjusted life years (Dalys) per year [4] with 90% of the current burden concentrated in Africa. It is endemic in more than 70 countries and territories in the tropics and subtropics and therefore remains a major public health problem worldwide [5]. *Schistosoma japonicum* is widespread in China and parts of South-East Asia [6]. In addition, it has been reported that more than 40 species of wild and domestic animals can be infected with *S. japonicum* [7,8]. The last decade has been marked by an extraordinary surge of awareness and funding for neglected tropical diseases, especially bilharzia. On a large scale, the fight against schistosomiasis has become more than ever the concern of sovereign States, guarantors of the health of populations, international institutions as well as humanitarian and non-profit organizations.

However, the number of people still needing treatment is not encouraging [9]. The increasing incidence of the disease seems to be leading governments to intensify health policies in the area of prevention and care. Thus, the overall objective of current public health strategies for schistosomiasis is to reduce morbidity through preventive chemotherapy (PC) [10]. Large-scale periodic administration of praziquantel, focusing in this case on the school-age population but also on adults living in high-risk (highly endemic) areas, aims to reduce the prevalence and intensity of infection [11]. However, its usefulness is sometimes limited enough in areas with high rates of reinfection and resistance. In addition, the low susceptibility of *Schistosoma mansoni* and *S. japonicum* to Praziquantel (PZQ) has been induced by mass drug administration programmes [5,12]. The strategies implemented to control bilharzia in West Africa have certainly contributed to a decrease in the incidence of the disease, although the challenges remain [12,13]. From an optimization perspective, it is necessary to take stock of these strategies, to evaluate their impact, their strength and weakness. This situation motivates the authors who are trying to identify and analyse the gaps in the knowledge which is intended to be filled with this review. In fact, they plan to enumerate the assets, to evaluate the strengths and weaknesses of previous studies and make objective proposals to motivate public authorities and organizations involved in the fight against bilharzia to refocus control strategies on an integrated approach (sensitization-chemotherapy-sensitization), to strengthen the sanitary and behavioral hygiene system through the construction of social and health infrastructures in endemic areas.

Indeed, the following study intends to fill up the gaps in the knowledge developed by the formers authors as far as the strategies used to fight against bilharziasis are concerned. This systematic review has to synthesize existing evidence on control strategies carried out by West African countries. It is supposed to inventory the means and techniques implemented and to study their limitations in order to develop a more adequate control plan capable of reducing or even definitively eradicating bilharzia from endemic countries. More over the meta-analysis aims to combine numerical data from multiple separate studies from the reviews, to capitalize on what has been learned, to analyze in a descriptive way the global level of the phenomenon (bilharziasis) and its variation in West Africa and finally to identify the nature of the interventions implemented. Therefore, it has a particular design to consider the summary of the studies concerned and the robustness of the conclusion and the recommendation of the meta analysis. This study was initiated as a systematic literature review and meta-analysis to identify lists of bilharziasis reviewer studies and presents as overall objective to develop a systematic literature review and meta-analysis of interventional studies conducted on urogenital bilharzia 2010-2021 in West African countries. Specifically the study aims to: produce a synthesis of control strategies carried out in West Africa from 2010 to 2021 and determine the effect of interventions carried out in the framework of schistosome control from 2010 to 2021 in West African countries.

## Methods

PRISMA guidelines [14] were used to conduct the systematic review. A literature search was conducted in the published literature (peer-reviewed journal articles) and grey literature (conference reports, theses and dissertations). Selected electronic databases including Google, Google Scholar, PubMed, Direct Science, Web of Sciences, PloS, Medline, and BDSP were targeted with appropriate keywords.

**Eligibility and ineligibility criteria:** a study is included if: it proposes a theme centred essentially on bilharziasis in general or urogenital bilharziasis in particular as well as anti-bilharzian control techniques (curative treatment and preventive control methods); it is taken into account by publications (journals and articles, theses and dissertations), in the period 2010 to 2020, and whose themes meet the expectations of the objectives of the systematic review and it is carried out in West Africa or sub-Saharan Africa. He was excluded from this study: studies on topics other than bilharzia; studies published in a language other than English, French; studies that represent just a narrative review; studies less than 2010 and those completed after 2020 and articles that do not accurately reflect the period of study.

**Search strategy:** the search was done in July 2021 and was conducted on scientific databases. Some Boolean operators were used to make the necessary interconnections to facilitate the selection of the best studies that best meet the journal writing. In accordance with the study question, a search equation was used in scientific databases. Almost all of the searches were done online and with syntax and keywords from the search theme. We decrypted results from a number of search engines, the main ones being PubMed, PloS, ScienceDirect, Web of Sciences, Google, Google Scholar and BDSP. Some Boolean operators were used to make the necessary interconnections to facilitate the selection of the best studies that best meet the editorial needs of the journal. The themes addressed in these different research areas concern NTDs in general and bilharzia in particular, as well as data from epidemiological studies. The research was later complemented by an epidemiological study done on bilharzia in Benin in 2020.

**Selection of studies:** the selection was made independently but on the basis of titles, abstracts and full texts, following the eligibility criteria listed above. The results were then pooled. Studies that may have been selected by only one or the other researcher were discussed for inclusion or rejection. Articles were included on the basis of the predefined selection criteria: publications addressing the targeted themes and published in French or English between 2010 and 2021; reports on estimates of the association between prevalence and the impact of socio-demographic and economic factors on the resurgence of bilharzia or the prevalence of the underlying infection, with a measure of statistical significance (e.g. p-value or 95% confidence interval) [15].

**Risk of bias assessment:** to avoid methodological errors and errors in the analysis of the data collected that would have systematic consequences on the quality of this literature review, biases were identified to assess the quality of the studies selected for the systematic review. These include confounding by poor analysis or by an incriminating factor that is not causally related to the study.

**Data extraction:** data extraction was done by simultaneous reading of the documents by the researchers. The data collected concerned the participants (number, age, gender, duration of the intervention, level of prosperity, socio-demographic factors) and the mode of transmission, anti-bilharzia strategies, inclusion criteria, and interventions (frequency, duration, types).

### Study variables

***Dependent variable:*** the dependent variable considered in the present study is the “prevalence” of the disease and in this case, it is represented by the number of positive and negative cases of bilharzia obtained before and after the interventions.

***Independent variables:*** regarding the independent variables “countries” and “years” are defined to make the analysis of the subgroups.

**Data processing and analysis:** the forest plot is used to highlight the overall effect of the interventions on the prevalence of bilharzia as well as the estimated effect size and confidence interval for each study. For this purpose, the Odd Ratio (OR) is the indicator used to measure the effect size with a 95% confidence interval (CI). Subgroup analyses were also performed by different characteristics such as country and study year. Heterogeneity and publication bias were captured through the I2 statistic and the Egger test. From the forest plot, the heterogeneity test was performed. The STATA 16 software was used for the analysis.

## Current status of knowledge

**Figure 1:**
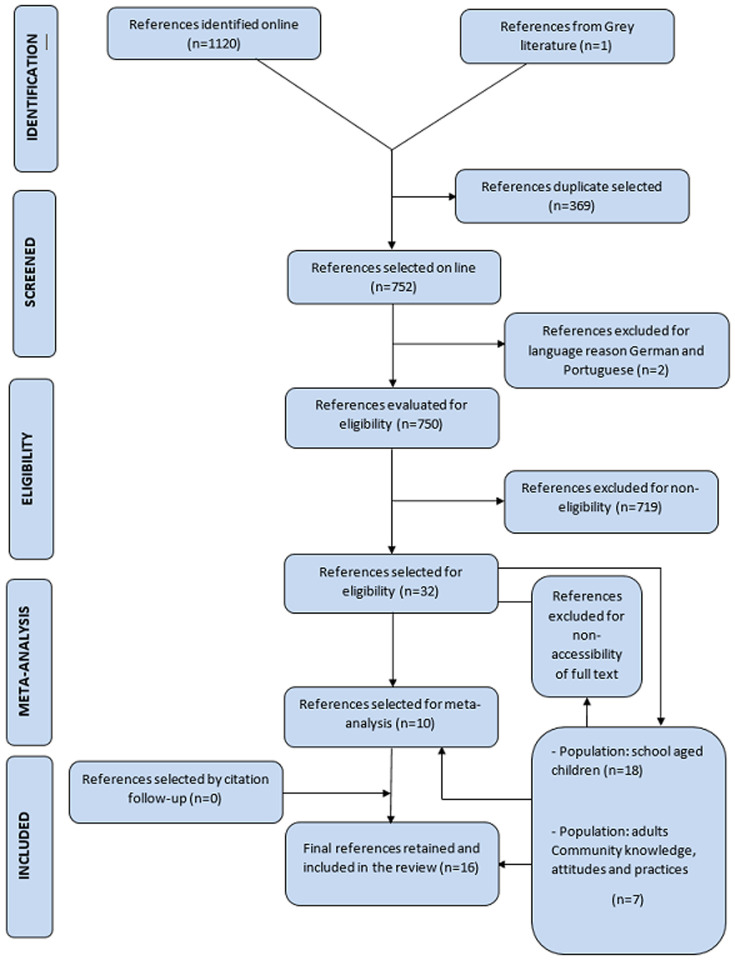
numbers of titles and studies reviewed in preparation of the current systematic review and meta-analysis bilharziasis and of chemical mollusciciding effects on Schistosoma-endemic area

**Selection of studies:** the selection of studies is summarized in the flow chart (Figure 1). Entering the search equation into the various search engines mentioned above yielded 1120 results. After removing duplicates, 752 studies were obtained. After reading the titles and abstracts and applying the eligibility criteria, 31 studies were retained. Sixteen references (Figure 1) were then excluded because the full text was not available. Thus, in the end, 16 articles were included in the systematic review (Figure 1).

***Source 1:*** compiled by the authors based on the processing of epidemiological data and interventions in the framework of bilharzia control in West Africa from 2010 to 2021.

**Data synthesis:** the works included in this review come from different countries in sub-Saharan Africa, such as: Benin, Ghana, Mali, Senegal, Burkina Faso, Sierra Leone and Niger. Four (04) studies were conducted in Mali, five (05) studies were conducted in Niger, two (02) studies were conducted in Benin and two (02) studies were conducted in Burkina Faso Table 1, Table 1 (suite), Table 1 (suite 1), Table 1 (suite 2) provides a summary of the articles reviewed.

**Table 1:** summary and characteristics of studies included in the systematic review

N	Authors and Year of Publication	Location (Country of study)	Type of study	Nature of the intervention/Strategy/ Treatment	Target (s) of the interventions	Duration of interventions	Impact of interventions	Prevalence measured before interventions	Prevalence measured after interventions	Proportions of prevalence reduction calculated	References
1	Yakuba Mr. Bah, Jusufu Paye, Mohamed S. 2019	In seven districts of (Sierra Leone)	Cohort and randomized study with a cross-sectional and longitudinal survey	Parasitological examination of 1980 stool samples and 1382 urine samples followed by CT of PZQ in 2009 and an evaluation in 2012	50 students (9 to 14 years old) randomly selected per school.	2-3cycles the3-6 cycles	Significant reduction of shistosomes (*S. haematobium* and *S. mansoni*)	42%	20.4%	20,4%	(13)
2	M Ibikounlé, A Ogouyèmi- Hounto, Y. Sissinto Savi de Tové *et al*. 2010	Péhunco in the North (Benin)	Parasitological and malacological survey	Prevalence study and malacological research	Children up to the age of 12 (58.27%).	Between May and September 2010 (5months)	Five species of molluscs are highlighted, two of which are known as potential intermediate hosts of bilharzia.	96%	59,22%	36%	(15)
3	Hamado Ouedraogo, François Drabo *et al*. 2013	Burkina Faso	National evaluation based on 22 randomized sentinel sites	Decade of biennial mass administration of praziquantel on schistosomiasis	Students aged 7 to 9 years	2004-2013	Massive use of preventive chemotherapy, which may have eliminated schistosomiasis as a public health problem in eight regions and controlled schistosomiasis-related morbidity in three other regions.	32,3%	82,6%	36,78%	(16)
4	Amadou Garba, Nouhou Barkiréc, Ali Djibo, 2010	Niger	Epidemiological, cross-sectional study with analytical purposes	Parasitological survey by filtration technique for 2 urine samples in consecutive days for *S. haematobium* and Kato Kartz for stool for diagnosis of S.mansoni followed by preventive chemoprophylaxis by CT and CAP study.	282 pre-school children and 224 mothers	The month of April	Schistosomiasis screening in a significant proportion of children in the 5-year age group	poorly known	An average of 45.85% for *S. mansoni* and 54,3% for *S. haematobium*	unknown	(17)

**Table 1 (suite):** summary and characteristics of studies included in the systematic review

N	Authors and Year of Publication	Location (Country of study)	Type of study	Nature of the intervention/Strategy/ Treatment	Target (s) of the interventions	Duration of interventions	Impact of interventions	Prevalence measured before interventions		Prevalence measured after interventions	Proportions of prevalence reduction calculated	References
5	Elias Asuming Brempong, Ben Gyan Abena Serwa Amoah *et al*. in January 2015	Ghana Town Of Pakro	Randomize d cross-sectional study	Parasitological study urine and stool sample collection followed by urine filtration, formalin ether concentration centrifugation method, sedimentation technique method and PCE ELISA method	308 participants aged 6 to 96	Six weeks	Existence of correlation between the levels of Cationic Eosinophil Proteins which are positively associated with the intensity of infection by the number of eggs in Schistosomiasis infections.	40%	59 (19.15%) Prevalence rate has no statistically significant relationship with frequency of contact with the water body (P=0.26).		19% (P=0,26).	(18)
6	Abdoulaye Dabo, Adama Z Diarra *et al*. 2015	Mali (Bamako)	Cross- sectional and cohort study	Parasitological study by Kato Kartz technique and urine filtration followed by 4 malacological explorations	Students aged 8 to 15 years	October 2011 and February 2012	High risk of schistosomiasis transmission in Bamako because it is present in the six municipalities	46.7% of *S. haematobium* and 28.2%of *S. mansoni* respectively in 1997	(14.7%) (n=1761) *S. haematobium* and (1.5%) (n=1491) *S. mansoni* Increase in January and February for B. pfeifferi and B.truncatu Respectively		32% in January and 26.7% in February 2012	(19)
7	Stefano Catalano, Elsa Léger, *et al*. In 2020	Senegal	Randomized study and malacological survey	Parasitological survey	Populations of 290 students in classroom situations	Between October and December 2017	Screening for multiple lineages of *S. mansoni* that can affect humans	On a local scale, diagnosis of S.mansoni infection ranged 3.8%-44.8% in school- aged children	52,6% 32%-40% for *S. mansoni* and 77%- 81% for *S. haematobium* and schistosome hybrids in school-aged children and adults		7,8%	(20)
8	Abdoulaye Dabo Mouctar Diallo Privat Koba Agniwo *et al*. 2021	Mali	A cross-sectional observational study	Mass treatment with PZQ (600mg)	1836 school-age children (7-14 years).	December 2014-2015 and April 2018	From 2014 to 2018, significant reduction in the prevalence of *S. haematobium* in some districts	6% with an alpha risk of 5%. We added 10% to this sample size	Increase of prevalence from zero to 96.8% in 2014; Decrease to 11% in 2015 and then decrease to 33,95 in 2018		62,85%	(21)

**Table 1 (suite 1):** summary and characteristics of studies included in the systematic review

N	Authors and Year of Publication		Location (Country of study)	Type of study	Nature of the intervention/Strategy/ Treatment	Target (s) of the interventions	Duration of interventions	Impact of interventions	Prevalence measured before interventions	Prevalence measured after interventions	Proportions of prevalence reduction calculated	References
9		Emily Y li, David gurarie, Nathan Clo *et al*. 2017	Africa	Comparative study of WHO guidelines since 2012 to an alternative adaptive decision-making framework for control in heterogeneous environment s to achieve defined public health goals.	CT at PZQ	WHO targets in children aged 5-14 years of less than 5% and less than 1%.	5-6 years of unsuccessful results	In the present study, the stratified worm burden modeling method, we examined whether the current guidelines for achieving key public health goals in low, moderate and high transmission communities.	CTs without permanent population sensitization effects do not lead to a significant reduction in the prevalence of bilharzia	Difficulty of coverage of CTs increased by 85% for children aged 5-14 years and by 40% for people aged 15 years and over. Low probability of reaching villages (prevalence <10% for children aged 5-14 years),	None	(22)
10		Anthony Danso- Appiah, Amadou Djirmay Garba *et al*. 2021	Countries in sub Saharan Africa	Systematic review and meta-analysis 1979 to 31 March 2021	PZQ CT at a single oral dose of 40, 50, 60, 70 or ≥80 mg/kg, and for dose comparisons of PZQ at 40 mg/kg.	School age children (5-14 years).	1979 to 2021 (42 years old)	Praziquantel reduced the prevalence of *S. haematobium* in school-aged children. For *S. mansoni*, there were reductions in prevalence at 12 months (RR 0.56, 95% CI 0.46 to 0.69;	The current cut-offs for CTs at PZQ are based On anecdotal evidence of prevalence in school-aged children. 50% by parasitological methods	The results showed a statistically significant reduction in the prevalence of infection at 12 months. prevalence of infection at 12 months	The 10% prevalence should be used as the “overall” prevalence and as a “global” threshold for the implementation of CT in endemic countries.	(23)
11		Bintou LY, Alpha Seydou YARO, Bernard Sodi *et al*. 2021	Mali	Observational and comparative epidemiological study of prevalence	Observational and comparative	School-age children (5-14 years).	Three years of treatment	Despite repeated mass treatment, complications resulted in significant kidney and bladder damage in male subjects.	Overall prevalence of 30%.	Low Infestation	20%	(24)
12		National Communicable Disease Control Programme (2013 to 2015)	Benin 77 communities in Benin	Epidemiological and randomized study	Parasitological investigations by urine filtration and Kato-Kartz Estimated treatment coverage population of 450277 or 15%.	19250 schoolchildren aged 8 to 14	Two years of treatment	Control schistosomes for the reduction of schistosomiasis to less than 10% in 75% of school-aged children by 2020	The prevalence of schistosomiasis at the national level is on average 20%. It varies from 0.40% to 91% depending on the commune	Prevalence greater than 50%), need for annual CT scan at Pzq Prevalence between 10 and 50, CT scan every two years. Prevalence between 0.4 and 10%, treatment at entry and exit of primary school	Ranges from 99.6% to 9%.	(25)

**Table 1 (suite 2):** summary and characteristics of studies included in the systematic review

N	Authors and Year of Publication	Location (Country of study)	Type of study	Nature of the intervention/Strategy/ Treatment	Target (s) of the interventions	Duration of interventions	Impact of interventions	Prevalence measured before interventions	Prevalence measured after interventions	Proportions of prevalence reduction calculated	References
13	A. Garba, S Toure, R Dembele *et al*. 2009	Burkina Faso, Mali and Niger	Randomized and cohort study	Parasitological examinations (urine filtrations and thick Kato-Katz smears) at each visit followed by CT scans of (2) 40 mg/kg oral doses of PZQ at 3 week intervals. Morbidity control. Cost-effectiveness study comparing a school-based and community-based strategy. Further study of the efficacy and safety of PZQ.	Infants and pre-school children with an average age of 2.6 years).	Three years of control; and 3 week treatment intervals	Reducing the prevalence rate	86% and 95%.	69% and 71%,	17% and 24%,	(26)
14	Amadou Garba Mariama S. Lamine Nouhou Barkiré *et al*. 2013	Niger	Analytical and observational cohort study	Parasitological examinations in urine filtrations and thick Kato-Katz smears at each visit followed by CT scans of (2) 40mg/kg oral doses of PZQ at 3 week intervals.	877 pre-school children	3 and 6 week follow-ups after CT	Observation of side effects.Cure rate of *S. haematobium* notable 3 weeks after in children who received the 2^nd^ dose PZQ is effective in 2 doses at short intervals on *S.haematobim* but less effective on *S. mansoni*	12.7% *S. haematobium* 38.5% *S. mansoni*	44,3%	46,9%	(27)
15	Amadou Garba, Mariama S. Lamine Ali Djibo *et al*. 2012	Niger	Analytical cohort study	Evaluation of efficacy and safety of PZQ by CT to study cure rate (CR) and egg reduction rate (ERR) defined as the proportion of individuals infected with *S. haematobium* or *S. mansoni* at baseline who became egg negative 6 weeks after the first dose; and determination of ERR 6 months after the first dose of PZQ.	877 pre-school children infested with either *S. mansoni* or *S.haematobium* or both.	3 weeks separately and a 3 and 6 week follow-up after CT	Adverse reactions after administration of (PZQ) (abdominal pain and bloody diarrhea) Syrup of PZQ is well tolerated by preschool children and has moderate and high efficacy against S. haematobium but considerably lower efficacy against *S. mansoni*.	At baseline the geometric mean (GM) infection intensity of *S. haematobium* ranged from 3.6 to 30.3 eggs/10 ml of urine (GM) of *S.mansoni* from 86.7 to 151.4 eggs/gr am of stool	49.2% to 100% reduction depending on the level of endemicity of the locality	94,9%	(28)
16	Mr. Bagaya, D Zongo B Savadogo, 2014	Burkina Faso	Random and coded sample	Malacological survey followed by examinations parasitological without CT	323 students in 2 schools	Malacological survey in 2011 and parasitological examination in January 2012, i.e. 24 months	Persistence of schistosomiasis but with decreasing prevalence due to CT after 2 years and urbanization	None	Prevalence of Molluscs 2,78%. Prevalence of schistosomiasis 5,66%	None.	(29)

**Impact of anti-bilharzia interventions or strategies:** data collected from the various articles show that bilharzia is one of the most important neglected tropical diseases (NTDs) in terms of morbidity and mortality. Epidemiological data from these papers show that the disease is endemic in many developing countries, affecting mostly children, farmers and women who are in frequent contact with waters that may harbour the intermediate host molluscs [16-30]. Millions of people worldwide are infected with different species of schistosomes [31].

The studies included in this systematic literature review were conducted in Benin, Ghana, Senegal, Mali, Burkina Faso, Sierra Leone and Niger. In all these studies, the interventions were related to the diagnosis of bilharzia in the urine and stool of the target population and treatment with praziquantel. In Benin, of the two studies included in this study, one was concerned with malacological research, i.e. mollusc intermediate hosts of the parasite before infecting humans [15]. The Kato Kartz method was the most widely used for diagnosis [26]. In Mali, a study also looked at the evolution of the disease in the population without any treatment [19]. *Schistosoma haematobium* and *Schistosoma mansoni* are the two most identified species in these studies [25].

It has been noted that any control measures for bilharzia should involve three major components: chemotherapy treatment, improvement of the health situation, vector control and health education. Addressing these components would help reduce transmission and reinfection by encouraging individuals to observe protective health behaviors [16,21]. Chemotherapy treatment was the most discussed component in this literature review. Three anti-bilharzia drugs are used for this purpose. Praziquantel is the main known antibilharzian. Data show that it is effective on all species of schistosome. Its cure rate varies from 80% to 100% [28]. Metrifonate is only effective on *S. haematobium* but resistance has been reported in Mali and Senegal [20,21,24]. Oxamniquine is active on *S. mansoni*. An experimental treatment with Epiquantel carried out in Niger has shown its efficacy, accompanied by undesirable effects, such as abdominal pain and bloody diarrhea in school children [28]. It is also important to emphasize that the strategies implemented for chemotherapy allow the targeting of interventions at the geographical level and at the level of risk groups. In general, all studies have shown that chemotherapy is the most effective means of controlling bilharzia because it has been shown to decrease the prevalence of bilharzia after the studies. These studies have also shown that praziquantel could be used by at-risk populations as a preventive treatment, accompanied by health education, especially that related to hygiene measures.

**Meta analysis of bilharzia interventions:** in order to optimize the results of the various bilharzia interventions over the study period, a meta-analysis of ten studies was conducted on all bilharzia interventions in West Africa.

**Figure 2:**
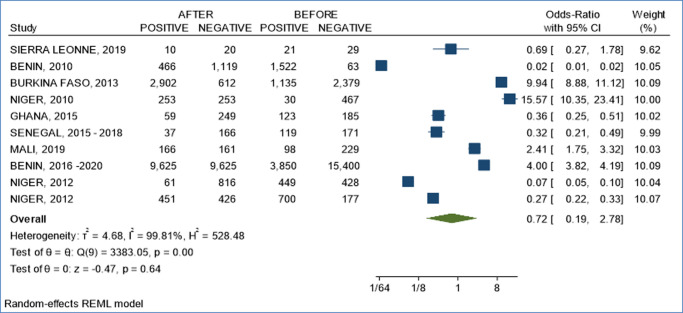
forest plot of the effect of interventions (chemotherapy through mass drug administration and community awareness) on bilharziasis studied as the phenomenon

**Forest plot of the effect of the intervention on the studied phenomenon:** Figure 2 presents the forest plot of the random effect model of the level of intervention on the studied phenomenon of bilharzia. There is a thematic correlation between all articles included in this study. All the works selected for this systematic literature review addressed the epidemiology, diagnostic methods and control measures (chemotherapy) of bilharzia in the study regions. There was no bias in the selected articles.

***Source 2:*** compiled by the authors based on the processing of epidemiological data and interventions in the framework of bilharzia control in West Africa from 2010 to 2021 (Figure 2).

**Figure 3:**
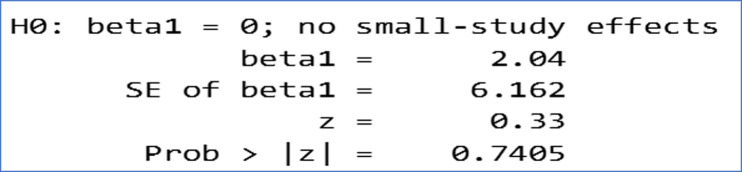
results of Egger's test for publication bias showing small study effects for the primary outcome (bias coefficient for the main analysis 95% confidence interval)

**Risk of bias:** Figure 3 presents the result of the Egger test for possible publication bias and shows that there is a homogeneity of studies with themes that respect the context of the systematic review (Figure 3). It has moreover demonstrated that the model is significant at the 5% level (p = 0.00) and the I2 statistic is 99.51% (Figure 3), which suggests strong heterogeneity. Similarly, the Egger test indicates the absence of publication bias (Prob > | Z | = 0.7405) (Figure 3). Thus, Figure 2 shows that there is no small sample size bias in this meta-analysis. Overall, the control interventions had a positive effect on the prevalence of bilharzia. Compared to the pre-intervention effects, an individual is 0.72 times less likely to contract the disease after intervention. However, some interventions had effects contrary to those normally expected. This trend was observed in Sierra Leone, Benin, Ghana, Senegal and Niger where a higher prevalence was observed after the intervention than before the intervention. Furthermore, the overall Odd-Ratio is 0.72 CI [0.19; 2.78] (Figure 3) which translates into a 0.72-fold lower risk of contracting bilharzia for an individual who has undergone the intervention compared to his or her counterpart living in an endemic area and not having undergone the intervention. The same trend was observed regardless of the country and year of study. The risk is higher respectively in Ghana in 2015, 15.57 CI [10.35; 23.41] (Figure 3) and Benin 2016-2020; 4.00 CI [3.82; 4.19] (Figure 3) while the lowest value is recorded in Benin in 2010 with 0.02 CI [0.01; 0.02] (Figure 3).

***Source 3:*** compiled by the authors based on the processing of epidemiological data and interventions in the framework of bilharzia control in West Africa from 2010 to 2021 (Figure 3).

**Figure 4:**
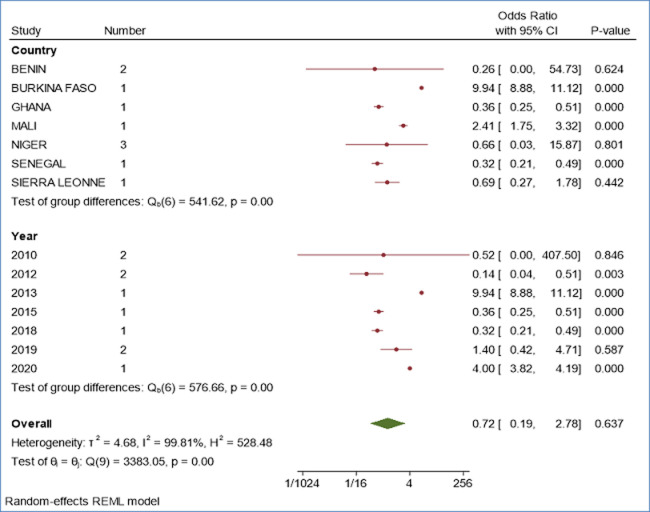
subgroup analysis of the effect of interventions on the phenomenon studied showing the effect of the intervention represented by Praziquantel Mass Treatment (PMT) on bilharzia

**Subgroup analysis of the effect of interventions on the phenomenon studied:** Figure 4 presents the subgroup analysis of the effect of the intervention, which here represents Praziquantel Mass Treatment (PMT), on reducing the number of people who contracted bilharzia in West Africa. Depending on the subgroup considered, we find both Odds-inferior to 1 for some values and superior to 1 for others and the p- value ratio is 0.000, which means that an individual who has undergone treatment is at less risk than one who has not received treatment. The overall trend observed is maintained within the subgroups and the extreme effect sizes are obtained in Burkina Faso (9.94; CI [8.88; 11.12]) (Figure 4) and Benin (0.26; CI [0.00; 54.73]) (Figure 4). Moreover, we also note a more or less significant progression of the odd with the lowest value observed in 2012 (0.14; CI [0.04; 0.51]) (Figure 4) and the value (4.00; CI [3.82; 4.19]) (Figure 4) obtained in 2020 with a peak of (9.94; CI [8.88; 11.12]) observed in the year 2013. This study therefore reveals that the prevalence of bilharzia has progressively increased from 2010 to 2020 which confirms that the risk of bilharzia contamination has remained constant throughout the last ten years with a very high level of endemicity. All West African countries considered in the study are affected by the disease and regardless of their geographical location, the odd ratio has increased significantly. When we look at the inter-group variations, we record fairly significant differences between countries, considering the effect of the interventions on the phenomenon studied on the one hand and the geographical location of the countries on the other (p = 0) (Figure 4). It follows that the effect of the interventions carried out on the phenomenon studied in West Africa did not remain constant over the study period considered (Figure 4).

***Source 4:*** compiled by the authors based on the processing of epidemiological data and interventions in the framework of bilharzia control in West Africa from 2010 to 2021 (Figure 4)

### Discussion

The aim of this systematic review was to synthesize existing evidence on the control strategies of West African countries against bilharzia. To ensure the quality of the methodology, the Prefered Reporting Items for Systematic reviews and Meta-Analyses (PRISMA) guideline criteria were followed at all stages of this systematic review. The main criterion was the situational analysis of the current situation through an assessment of control strategies and prospects for the eradication of bilharzia [32].

In general, health outcomes are unevenly distributed. In fact, several determinants have a considerable influence on the occurrence and recrudescence of bilharzia. These include behavioural hygiene, the availability of socio-community infrastructure (access to drinking water and sanitation facilities), environmental hygiene, exposure through working conditions by considering the case of populations forced to use waterways in the exercise of their fishing or farming profession and access to health services. As a result, this disease systematically hinders school attendance and performance.

The analysis of the data collected from the articles made it possible to identify several studies carried out in West African countries. These include Benin, Niger, Mali, Burkina Faso, Senegal, and Ghana [16,20,21,32]. These regions represent endemic areas for the occurrence of bilharzia. The prevalence study carried out shows a very high incidence of schistosomiasis by mass diagnostic surveys [13,15,17,18,20,25]. The African regions constitute indeed zones of predilection of the intermediate hosts of *S. haematobium* and *S. mansoni* [33]. The control strategies were essentially the identification of intermediate hosts and chemotherapy with PZQ. [22,25,30,31]. Currently mass drug administration campaigns remain the most studied and used strategy according to WHO guidelines [1,3,12].

The most studied target populations are school children under 12 years of age. The management of bilharziasis focuses on preventive and curative treatment [20]. Biannual treatment with praziquantel is one of the effective control strategies for bilharzia [34]. Anti-infective treatment has been shown to significantly reduce the parasite load of *S. mansoni*, but this is not always the case for *S. haematobium* in infested children under 6 years of age [21]. In West Africa, despite these measures, the control of schistosomiasis becomes complicated because *Schistosoma spp*. is capable of infecting several definitive hosts [20]. Vector control is therefore becoming a strategy of choice in the fight against schistosomiasis in the face of the many environmental changes that African countries are witnessing [19]. Drinking water supply is an important strategy in pest control, as bilharzia vectors have water as their natural habitat. Riverine populations without access to safe water are therefore at much greater risk of the disease [19,35]. As noted in several cohort studies, when shellfish control is combined with population-based screening and selective or mass drug therapy, prevalence would be reduced more rapidly and incidence would decrease in the population [21,36]. However, transmission was often not eliminated. Successful shellfish control programmes have significantly reduced the local prevalence of Schistosome infection [37]. But some control programmes have had minimal impact on local prevalence [33]. Although there were some apparent differences in effects by region and parasite species, the mollusc species identified in the included studies were too diverse to make meaningful comparisons for prevalence or incidence results stratified at the intermediate host species level [36].

According to the standard control rule, children aged 5-14 years have a 50% or greater chance of being infected after preventive chemotherapy performed once a year [22]. Thus, the basic control strategy for bilharzia is to choose the initial frequency of treatment based on the baseline prevalence and then re-evaluate after 5-6 years of the same drug. Depending on the prevalence of infection, the community is then given a possible change in the frequency of chemotherapy administration. The latter is re-evaluated after an additional 4 years of treatment to see if the objectives of the program have been achieved beacause the rate of infection has dropped considerably. Indeed, the objective of this control strategy is to achieve a morbidity of less than 5% of prevalence of heavy infection in children aged 5 to 14 years or the objective of eliminating less than 1% of prevalence of heavy infection [12,22]. A study carried out in 2021 in Mali on the massive distribution of drugs to school children reported a decrease in *S. haematobium* infection, however, the WHO requires that praziquantel not be used in preventive chemotherapy campaigns in school children because, to date, there is no suitable formulation. [1]. It would therefore be appropriate to re-evaluate the control of schistosomiasis in children with praziquantel.

In fact, the meta-analysis showed that overall, the interventions had a positive effect on bilharzia because of the way it spreads and its extent in the world, it is considered as a public health problem that undoubtedly impacts economic and socio-demographic conditions in a world development. Furthermore, it is an evidence that bilharzia is one of the neglected tropical diseases targeted by the WHO with endemic areas where the prevalence has reached alarming proportions [38,39]. The results indicate that the interventions carried out in the framework of the control of bilharzia with PZQ have an effect on the reduction of the prevalence in several countries whatever the country and the period of study. Indeed, the risk of bilharzia infection is reduced when the subject undergoes a preventive intervention (administration of praziquantel). However, the risk could be further reduced when the administration of PZQ is accompanied by multiple household sensitization on water use and relations as well as the promotion of socio-sanitary infrastructures.

In addition, according to WHO, current strategies for schistosomiasis control aim to prevent morbidity in later life through regular population monitoring by mass drug administration to at-risk populations in so-called homogeneous ecological zones [40,41]. This confirms the importance of the interventions implemented on both sides in the studies reviewed for the meta-analysis. However, according to studies conducted by Sturrock *et al*. it is clear that Mass Drug Treatment (MDT) alone is not the only solution to reduce the spread of schistosomiasis and therefore additional interventions are needed and should be implemented [40].

These results are consistent with previous studies by Danso-Appiah *et al*. [23] on the threshold prevalence observed during a MDT in May 2021 which showed that when Praziquantel is administered, the prevalence of *S. mansoni* and *S. haematobium* in school children decreases, but with no apparent difference between baseline and forty-eight months of treatment. The same is true for annual MDT administration for *S. mansoni*, which resulted in a reduction in children and adults, but no apparent reduction after twelve months of repeated MDT. At the community level, treatment has produced similar results with annual mass drug administration for nine years, but this has not reduced prevalence to the target of elimination of *S. mansoni* and *S. haematobium* in settings where baseline prevalence was 10% or more [31].

A malacological study carried out in the town of Péhunko in Benin in 2014 has shown results [15] much lower than those obtained by Ibikounlé *et al*. in 2009 [31,41] with a high prevalence of the condition (96%) in the village of Doh alone with a Z statistical test of the Stat View software (Z=2.555; p>0.05). It appears that this decrease in the infestation rate could be linked to the sensitization of the population and the mass treatments with praziquantel organized in the commune [42-44]. While the control strategy for schistosomes is preferably characterized by a mass survey followed by the administration of Praziquantel to communities, the use of molluscicides is also a control strategy recommended by the World Health Organization for the development of effective and practical measures for the control of schistosomiasis through snail elimination.

In this perspective, King *et al*. in a non-randomized study conducted in the United States in November 2015 under the control of the Center for Global Health and Diseases and the Schistosomiasis Consortium for Operational Research and Evaluation (SCORE) [35] had found that there is a positive correlation between the use of molluscicide in the control of snails and mass treatments with praziquantel. Thus, according to its authors, this synergy in the bilharzia control strategy is an effective method of reducing Schistosoma infections over time, with an additive effect on prevalence when population-based drug control is also carried out [45,46].

On the other hand, Knopp *et al*. in a study carried out in April 2013 in Zanzibar went further by highlighting that in the context of school-based treatment programmes, only the implementation of triple therapy consisting of snail control by molluscicides, preventive chemotherapy campaigns, and probably also the improvement of sanitary infrastructures, have reduced the prevalences of schistosomiasis from very high levels (50%) in the 1980s to a low level today [43-46]. It is therefore obvious that according to the authors, the reduction of the prevalence cannot only be the prerogative of a MDT with PZQ, nor the use of molluscicides against the hosts hosting miracidium, and that it is necessary to closely associate the teaching of hygiene notions by multiple sensitization sessions for a change of behaviour.

The results obtained from the present study deserve to be used taking into account some limitations common to schistosome control interventions. In general, however, it should be noted that the interventions identified are aimed to reduce the prevalence of schistosomiasis in endemic areas. Nevertheless, the resurgence and re-emergence of the disease despite the types of interventions commonly implemented seem to indicate the inadequacy of the strategies used in schistosomiasis control. It appears that raising awareness of good hygiene practices to encourage behavioural change and complementing traditional control strategies would be the most important means of accelerating the reduction of the prevalence rate. This study is innovative with its major assets being the elaboration of a synthetic view of the control strategies implemented in the fight against schistosomiasis, to bring out the positive effects and the impact of the treatments administered to the populations and to make a comparison of the interventions carried out in the different countries of West Africa.

**Limitations:** the major limitation is the very low number of studies of an experimental nature highlighting comparative data before and after interventions which does not favoring a broad-spectrum comparison. We therefore suggest that anti-schistosomiasis control strategies be more multispectral and carried out in a context of interventions including all the main actors such as religious, community leaders, traditional chiefs and in particular public authorities. Each intervention must be carried out in the context of evaluation **"before and after intervention"**so as to map the impacts in order to hope for better results tending to the elimination of schistosomiasis.

## Conclusion

Annual antiparasitic distribution campaigns remain the most widely used control strategy, however, host-vector and human-to-human transmission related to poor hygiene result in a continuous rebound in infection prevalence. Repeated implementation of annual mass distribution campaigns based on WHO guidelines allows for an initial reduction in prevalence in two to three rounds. However, subsequent rounds do not allow for further reduction in prevalence of infection, which has prevented the elimination of the public health problem. Uncertainties regarding aspects of human and shellfish biology and exposure factors must be considered for effective control. It would therefore be important to encourage vector control in schistosomiasis control strategies.

### What is known about this topic

- *In the medical community the bilharziasis disease is sufficiently well known;*
- *Bilharziasis is well known as an NTD disease commonly in endemic area;*
- *The bilharziasis disease is known to be cured by the administration of PZQ*.

### What this study adds

- *This study evaluates the strengths and weaknesses of the strategies developed to fight against bilharziasis;*
- *The study notes the inadequacies of the implemented control strategies;*
- *This study is initiated to highlight the implementing of an integrated control strategy against bilharziasis for better results*.
